# Relationship between the Fungal Incidence, Water Activity, Humidity, and Aflatoxin Content in Maize Samples from the Highlands and Coast of Ecuador

**DOI:** 10.3390/toxins14030196

**Published:** 2022-03-06

**Authors:** Héctor Abel Palacios, Andrieli Stefanello, Margarita Susana García Gavilánez, Dicke Alejandro Castro Demera, Marcelo Valle Garcia, Wilson Arturo Vásquez Castillo, Marcelo Alejandro Almeida Marcano, Iván Rodrigo Samaniego Maigua, Marina Venturini Copetti

**Affiliations:** 1 Facultad de Ingeniería Mecánica y Ciencias de la Producción, Escuela Superior Politécnica del Litoral, ESPOL, Guayaquil 09-01.5863, Ecuador; 2 Facultad de Ingeniería Agroindustrial, Universidad de las Américas, UDLA, Quito 170513, Ecuador; margarita.susana@gmail.com (M.S.G.G.); Alejocastro2701@gmail.com (D.A.C.D.); wilson.vasquez@udla.edu.ec (W.A.V.C.); marcelo.almeida.marcano@udla.edu.ec (M.A.A.M.); 3 Graduate Program of Food Science and Technology (PPGCTA), Universidade Federal de Santa Maria, UFSM, Santa Maria 97105-900, RS, Brazil; andri_stefanello@hotmail.com; 4 Latis Scientific, Dartford DA1 4AL, UK; marcelo.unipa@gmail.com; 5 Instituto Nacional de Investigaciones Agropecuarias, INIAP, Cutuglahua 171107, Ecuador; ivan.samaniego@iniap.gob.ec

**Keywords:** maize, mycotoxins, aflatoxins, *Aspergillus flavus*

## Abstract

This study evaluated the fungal incidence through direct plating in Agar Dichloran Glycerol, and the presence of aflatoxins in maize samples from the Highlands and Coast of Ecuador by HPLC, investigating the influence of the temperature, altitude, water activity, and humidity of the collection regions on the maize samples’ contamination using Principal Components Analysis (PCA). The overall kernel infection by fungi was usually lower in samples from the Highlands, and no aflatoxins or *Aspergillus* series *Flavi* were detected in the samples from this region. In the coastal samples, *Aspergillus* sp. were isolated from all samples, while the potentially aflatoxigenic *A. Flavi* contaminated about 80% of them. Aflatoxins were present in 50% of these samples, in ranges from 0.42 to 107.69 µg/kg. PCA was able to segregate the samples according to their collection region, and showed that the maximum and minimum temperatures are closely and positively related to the presence of *A. Flavi*. A highly positive relationship was also observed between the water activity of the sample and aflatoxin contamination. On the other hand, the altitude had a very strong—but negative—relationship with the variables studied. This study is relevant because data regarding fungi and aflatoxin occurrence, as well the main factor influencing the contamination of Ecuadoran maize, are scarce; it clearly shows that aflatoxins are a hazard present in maize from the Ecuadorian Coast but not the Highlands.

## 1. Introduction

According to the National Institute of Agricultural Research of Ecuador (INIAP), maize (*Zea mays* L.) is one of the country’s most important agricultural products in terms of the social economy, as it constitutes the main input for the elaboration of concentrated foodstuffs destined for the animal industry, especially for commercial poultry, and it represents a basic component of the diet of the rural population as a fundamental part of food security [1]. Although the cultivation of maize is not native to Ecuador, the country has different varieties that have been adapted to different altitudes. Among them, one finds “duro” (hard) maize and “suave” (soft) maize, which cover the largest amount of planted area on the Ecuadorian Coast and in the Highlands, respectively [2].

Like any other agricultural crop, maize can be affected by fungi; these micro-organisms are responsible for an important part of the diseases that affect this crop. Fungal species can occur at different stages of maize production, and are even related to spoilage problems occurring in the storage of kernels [3]. Rainfall, drought stress, and the use of maize cultivars which are not adapted to the cultivation region at hand are some factors that can influence the plant or kernel infection and facilitate the subsequent proliferation of toxigenic fungi, leading to the production of mycotoxins [4,5]. South America is predominantly a tropical and subtropical continent; it provides favorable environmental conditions for fungal growth on food crops, especially species such as *Aspergillus flavus (A. flavus*) and *Aspergillus parasiticus (A. parasiticus)*, which belong to the Series *Flavi* together with other similar and less common species [6]. Depending on the grain and weather conditions, high levels of aflatoxins (AFLs) can be produced during harvesting or storage [7]. Due to its geographical position, Ecuador presents a low fluctuation in temperatures and solar incidence throughout the year; however, strong differences in altitude, temperature, and humidity have been observed in the country’s Highland and Coastal regions due to their peculiar microclimates [8].

There is no accurate information in Ecuador about the quality of maize stored in silos [9]. If it existed, this could give a clear indication of the percentage of maize contaminated by mycotoxins in the nation’s provinces. Previous studies have demonstrated the prevalence of *Aspergillus* spp. in some maize varieties stored in silos, and of the subsequent production of secondary metabolites, such as aflatoxins [10,11]. Nonetheless, no studies showing the relationship between fungal contamination, aflatoxin content, and biotic factors on maize from Ecuador were found.

Therefore, the objective of this study was to quantify and identify the fungal incidence and presence of aflatoxins in maize samples from two different regions of Ecuador (the Highlands and the Coast) by evaluating the influence of abiotic factors, such as the temperature, altitude, water activity (WA) content, and humidity patterns of the regions where the samples were collected in order to assess the fungal and aflatoxin contamination of the maize samples.

## 2. Results and Discussion

Table 1 shows the aflatoxin and WA content, as well as the fungal infection and incidence rates on maize samples from the Highlands and Coast of Ecuador. In general, the kernel infection by fungi was usually lower in samples from the Highlands.

*Fusarium* sp. was the dominant genus present in 60% of the Highlands samples, followed by xerophilic *Aspergillus* (formerly *Eurotium* sp.) species. The only potentially toxigenic *Aspergillus* species present in the samples from this region were from the *Aspergillus niger* complex (16.7%), in only one sample. This species is ochratoxin A, and fumonisin B2 is a potential producer [12,13]. On the other hand, in the Coastal samples, the genus *Aspergillus* was isolated from all of the evaluated samples, where the presence of the potential aflatoxigenic *A. Flavi* was very common, occurring in 13 out of 16 samples. *Fusarium* sp. was also a major toxigenic genus, given that it was detected in 15 of the 16 samples.

Gasperini et al. [14] reported a similar fungal diversity to that found in our study when accessing the dominant fungal genera in 12 different maize cultivars from Brazil. The main genera found were *Fusarium*, *Penicillium*, *Aspergillus,* and occasionally *Cladosporium* and *Alternaria*. The strains of *A. flavus* isolated from those samples were potentially producers of aflatoxin B1. However, the authors did not detect aflatoxin in the maize samples, mainly due to the good storage conditions used for these grains. The predominance of the genera *Aspergillus* and *Fusarium* in maize is widely reported [12].

No aflatoxins, nor the presence of *A. flavus,* were detected in the samples from the Highlands. In contrast, seven out of 16 samples (43.7%) from the Coast were contaminated with aflatoxins, which also presented higher WA values (ranging from 0.57 to 0.90). Furthermore, in all of the samples where aflatoxins were detected, the presence of *A. flavus* was also confirmed. However, in some samples contaminated by *A. flavus,* aflatoxins were not detected.

Spread worldwide, *A. flavus* is the most common producer of aflatoxin B1 and B2 [6,12], and was possibly the only potential aflatoxigenic species isolated from the Ecuadorian maize samples evaluated here. These metabolites, which are considered highly toxic to humans and animals, are often found in maize samples [15,16]. However, the production of these metabolites will depend on certain relevant factors, such as the temperature, pH, substrate, relative humidity, drought stress, and the presence of other fungi [5,15,16]. This could justify the absence of aflatoxins in some samples where *A. flavus* was detected, because the conditions allowing fungal growth are wider than those required for aflatoxin production [12]. Furthermore, only about half of the *A. flavus* isolates were able to produce aflatoxins [12]. Jayaratne et al. [17] also described the importance of the soil quality. In their study, it was observed that high concentrations of aflatoxin B1 in the soil lead to an increase of this toxic metabolite in maize kernels, as the spores of toxigenic strains could be translocated to plants through wind and splashes.

The total aflatoxins on the contaminated samples ranged from 0.42 to 107.7 µg/kg, with an absence of aflatoxin G2 in all of the samples evaluated. Interestingly, the sample with the lowest WA content (0.57) showed the highest contamination rate of *A. flavus* (25%). On the other hand, the highest content of aflatoxins was detected in the sample with the highest WA rates (0.90) (Table 1).

Fungi acquired in the field—such as *Fusarium verticillioides, F. proliferatum, F. oxysporum,* and *A. flavus*—can persist in stored maize; together with their toxins, they can be carried through to maize products, thereby reaching consumers [12]. This could explain the highest level of aflatoxin detected in a low-WA sample. Broadly speaking, it is also important to highlight the distinct ecophysiology of those two genera, especially the WA required for growth, whereby *Fusarium* spp. usually interrupt their multiplication at post-harvest stages, while most *Aspergillus* spp. can still multiplicate during storage if the WA is above 0.75, or even because of the humidity changing due to the huge temperature variations in situations of poor storage in silos.

In a study performed by Vásquez & Vásquez [9], the presence of *Aspergillus* spp. and its main metabolite (i.e., aflatoxin) were investigated in three storage silos from Ecuador. The aflatoxin values reported in that study exceeded those indicated by national (INEN) and international (CODEX) regulations, which are 20 ppb in Ecuador, and the same value in China and Brazil [18,19]. Nevertheless, the European Union’s limit is more restricted for aflatoxin B1 in maize, with these being 5 ppb for processing products and 2 ppb for direct consumption [20]. In the United States, the permitted limit for total aflatoxin is 20 ppb [21]

Within the 28 samples analyzed in our study, only eight showed aflatoxin contamination, representing 28.6%. According to a survey carried out by Biomin [22], from 3839 maize samples analyzed in Central America in 2020, only 12% had aflatoxin, with a maximum of 179 ppb. In contrast, the incidence of fumonisins was higher in these samples: out of the 3577 samples analyzed, 84% were contaminated with this metabolite, reaching a maximum value of 56,000 ppb. In our study, the quantification of fumonisins was not performed; however, the presence of *Fusarium* sp. was identified in eight out of 12 samples from the Highlands, and 15 out of 16 samples from the Coast.

The aflatoxin B1 levels in maize samples from the Highlands were below the quantification limit; the same was observed in eight samples from the Coast. However, in the remaining eight samples from the Coast, the aflatoxin B1 values ranged from 0.42 to 97.75 µg/kg. Similar results were found by [17] in 60 maize samples from the Anuradhapura district in Sri Lanka, 15 samples exceeded the acceptable levels of aflatoxin B1, while 23 samples were within the acceptable range, and 22 were aflatoxin-free. On the other hand, a study carried out by Kaaya et al. [23] also revealed that maize samples from the Highlands of Uganda were less contaminated by aflatoxins.

**Figure 1:**
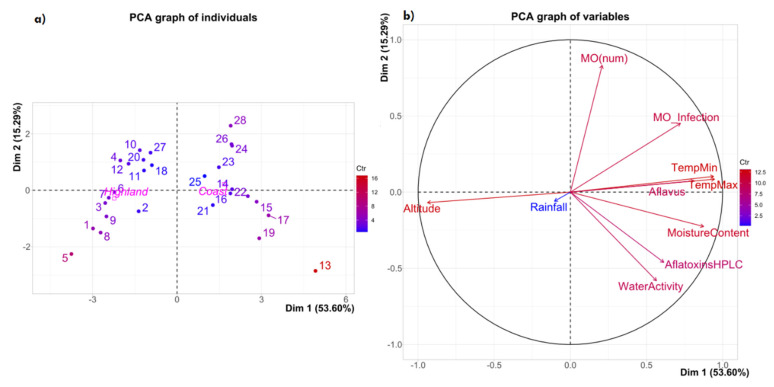
Principal components analysis of dimensions 1 and 2, showing the variability (68.89%) of the ten variables studied in maize samples from 14 localities in two regions of Ecuador: (**a**) individuals; (**b**) variables.

**Figure 2:**
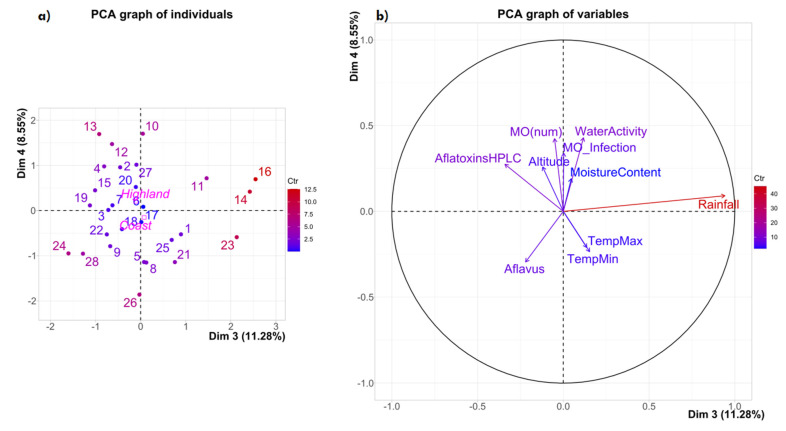
Principal components analysis of dimensions 3 and 4, showing the variability (19.83%) of the ten variables studied in the maize samples from 14 localities in two regions of Ecuador: (**a**) individuals; (**b**) variables.

The relationship between the characteristics of the samples’ regions of origin (altitude, minimal and maximum temperature, and rainfall) and the characteristics of the samples (WA, humidity, fungal contamination, and aflatoxin content) is shown in Figure 1 and Figure 2.

Considering all of the variables studied in the 14 localities where the samples were collected, two groups of maize can be clearly segregated according to the region of production. Those in quadrants 1 and 4 correspond to the Coastal samples, while those in quadrants 2 and 3 are from the inter-Andean valleys of Ecuador, i.e., the Highlands (Figure 1a). Within each region, it is possible to see groups of maize with great similarity. For instance, sample 13—from Balzar province in Guayas (the Coast)—was taken very far away from the other Coastal samples. This could be due to the combination of the high values of the following parameters: WA, grain moisture, aflatoxin, and the presence of *A. Flavi*. No aflatoxins were present in the other maize sample from the same locality. These differences are possibly related to failures in post-harvest drying, whereby raised humidity allowed for fungal growth and aflatoxin production. It is also important to mention that samples 25, 14, 22, 16, and 21 are closely related—in a positive way—to component 1, while samples 6, 7, and 3 are very similar, and are related to component 1, but in a negative manner.

In Figure 1b, it can be observed that the maximum and minimum temperatures are closely and positively related to the presence of *A. flavus*, which is one of the variables that best explains the variability in the first component (53.6%). It is also important to highlight the highly positive relationship between the samples’ WA and aflatoxin contamination. On the other hand, the altitude of the production sites has a very strong relationship with the first component, but is negatively related with all of the other variables studied. The variable that specifies the number of maize grains colonized by fungi is positively related to the second main component, which explains 15.3% of the variability present. It is vital to mention that, according to Figure 1b, rainfall has very little impact on the number of microorganisms that colonize the maize kernels. Finally, it should be noted that all of the variables analyzed in the maize samples are negatively related to altitude if dimension 1 is considered.

The dispersion of the samples (individuals) in dimensions 3 and 4 revealed two groups: group 1 consists of samples 11, 14, 23, and 16, while group 2 is comprised of the rest of the samples (Figure 2).

The results of PCA 3 and 4 explain around 20% of the existing variability of the variables studied (Figure 2a,b); however, they are less relevant than those which were presented in PCA 1 and 2 (Figure 1a,b), which explained 68.9%. Rainfall is the variable that is most related to component 3, and it has the greatest contrast, explaining the variability. Again, in this component, precipitation contrasts with *A. flavus,* the most common aflatoxin producer species in maize and also with the amount of aflatoxins in the kernels, and the altitude of the localities. Meanwhile, when considering component 4, a contrast is observed among the aflatoxins, altitude, general fungal contamination, WA, and moisture content against temperature and *A. flavus*.

## 3. Conclusions

This study evaluated the influence of different parameters, such as rainfall, altitude, maize variety, moisture, and WA content on the occurrence of *Aspergillus* species, with a focus on the potentially aflatoxigenic fungi and aflatoxin content of maize samples. The differences between the profile of *Aspergillus* series *Flavi* and aflatoxin occurrence were observed between samples from the Ecuadorian Highlands (soft maize), where this species and aflatoxins were absent, and the Coast (hard maize), where, respectively, 81.5% and 50% of the samples were infected by species potentially producers of aflatoxins from *Aspergillus* series *Flavi* and contaminated with aflatoxins.

## 4. Materials and Methods

### 4.1. Samples

The sampling methodology of the INEN 1233:95 standard was used, with slight modifications, which were subject to the specific harvest and post-harvest practices of each sampled location [24]. The collection of samples from the “duro” (hard) variety was carried out in different localities of the coastal provinces where the highest production of hard maize in Ecuador can be found, namely Guayas, Manabí, Los Ríos, and Loja. Furthermore, samples of the “suave” (soft) variety were taken in the Highland provinces of Azuay, Cotopaxi, Bolívar, Chimborazo, Pichincha, Carchi, and Tungurahua.

The maize samples were taken in bulk from 50 producers. They were collected in collection centers in every locality in each region. The localities studied are representative of each region’s maize production. In other words, the 28 composite corn samples studied represent 1153 producers.

### 4.2. Humidity Content

The principle of capacitance was used to determine the moisture content in the samples [25]. The method was executed with AgraTronixTM MT-16 Grain Humidity Taster (Portage, OH, USA) equipment, which has a high-frequency capacitance circuit that improves the precision of the data, giving automatic results in the determination of the moisture of the maize in a range between 5 and 40% [26]. The tests were carried out at a temperature between 22 and 24 °C.

### 4.3. Water Activity (WA)

Three subsamples of each maize sample had their WA directly quantified at 25 °C (±0.2 °C) using an electronic dew-point Aqualab 4TE WA meter (Decagon Devices, Pullman, Washington, DC, USA). In order to speed up the measurement time, the maize samples were placed in plastic sample holders and equilibrated at 25 °C in a DOB chamber.

### 4.4. Altitude, Temperature and Rainfall Data

Data on the altitude, relative humidity, maximum and minimum temperature, and precipitation of the places where the samples originated from were obtained from the National Institute of Meteorology and Hydrology of Ecuador (INHAMI).

### 4.5. Fungal Contamination and Identification

Initially, the maize kernels were disinfected by immersion in a 0.4% sodium hypochlorite solution for 1 min. A total of 24 grains were placed in 3 Petri dishes containing Dicloran Glycerol Agar 18% (DG18) with chloramphenicol. The dishes were incubated at 25 °C for 7 days. After the incubation period, the plates were examined, the kernels that showed fungal growth were counted, and the results were expressed as a percentage of infected kernels per sample, according to the methodology proposed by Pitt and Hocking [12].

The fungi were first isolated on Petri dishes containing Czapek Yeast Extract Agar (CYA) to be identified through specific protocols for each genus, calculating their frequency of occurrence in each sample.

*Aspergillus* spp. were inoculated at three points in the CYA and Malt Extract Agar (MEA) dishes, and were incubated for 7 days at 25 °C. In parallel, CYA dishes were inoculated with the same isolates, and were incubated at 37 °C for the same period. Xerophilic *Aspergillus* isolates belonging to the subgenus *Aspergillus* (formerly *Eurotium*) were additionally grown on Czapek Yeast with 20% Sucrose (CY20S) for 14 days at 25 °C.

Isolates of the *Penicillium* genus were inoculated at three points on Petri dishes containing CYA, MEA, Yeast Extract Sucrose Agar (YESA), and Creatine Yeast Extract Agar (CREA), and were incubated for 7 days at 25 °C. At the same time, CYA dishes were inoculated with the same isolates, and were incubated at 5 and 37 °C, for the same period.

Other miscellaneous genera were cultivated in CYA, MEA, and Clavate Leaf Agar (CLA) at 25° C for posterior identification.

After the incubation period, the species were identified according to the references indicated for each genus through the observation of the macro and microscopic characteristics of the colonies (such as the colony size, verse and reverse coloring, texture, exudate production and soluble pigment, shape, and the ornamentation of the microscopic structures). Finally, the frequency of occurrence for each species was calculated based on the total fungus presence in each sample.

The references used for the identification of the *Aspergillus* isolates were [13,26,27,28,29]. For the genus *Penicillium* (subgenus *Penicillium*) [30] and for the other subgenera [31] were used. The other miscellaneous fungal genera were identified according to the descriptions of [12], complemented with other sources if necessary.

### 4.6. Aflatoxins Analyses

For the quantification of the aflatoxins in the maize samples, the methodology proposed by Maigua et al. [32] was used, with modifications. Artificially contaminated samples with aflatoxins B1, B2, G1, and G2 in three different concentrations were used, and we evaluated the linearity of the method; the precision, by means of a repeatability test; and the accuracy, by means of a recovery test.

For this step, 50 g of the maize sample, previously powdered, was weighed and liquefied by adding 100 mL methanol: water solution (80:20, *v*/*v*) for three minutes at high speed. Then, it was centrifuged for 10 min at 4000 rpm and filtered using filter paper and glass fiber membrane. Then, 2 mL aliquots of the filtrate were taken and diluted with 25 mL phosphate-buffered saline (PBS).

The dilution obtained from the previous step was purified on immunoaffinity columns at a flow rate of 2 to 3 mL/min. The column was washed with 20 mL PBS, and the toxins were eluted from the column with 3 mL high-performance liquid chromatography (HPLC)-grade methanol.

This step was carried out in an HPLC system (Agilent technologies 1100/1200 series. Waldbronn, Germany) consisting of a binary pump (G1312A), a fluorescence detector (FLD G1321A) (λ excitation 362 nm and λ emission 425 nm), an auto-injector (G1329A), and a KOBRA CELL type post-column electrochemical derivatiser (R-Biopham Rhone. Ltd., Glasgow, Scotland), controlled by Chemstation software (Agilent Technologies, Waldbronn, Germany).

For this process, a separation process was performed using a C18 reversed-phase column. For its determination, 20 µL of the extract was added to the column at a flow rate of 1 mL/min. The mobile phase consisted of a mixture of water/methanol/acetonitrile with 119 mg potassium bromide and 350 μL 4 M nitric acid. For quantification, the areas obtained were compared on a calibration curve. The linearity of the method was evaluated by relating the response function of the equipment with the concentration of certified aflatoxin standards.

For the elaboration, a stock solution of 2500 μg/mL was prepared by adding 4 mL toluene:acetonitrile solution to vials that contained 10 mg of each aflatoxin. This was stirred until it was completely diluted, and then stored at −20 °C. The aflatoxin standards B1, B2, G1, and G2 had 98% purity, and were obtained from Sigma Aldrich (St. Louis, MO, USA). The standard working (2500 ng/mL) and the calibration solution (10 ng/mL) were prepared from the dilution of the stock solution in HPLC-grade methanol, and were stored at −20 °C.

Purified water with a conductivity of 18.2 MΩ was used throughout the procedure (Cm at 25 °C). The reagents—sodium chloride, sodium hydroxide (≥99%), potassium bromide (≥99%), potassium chloride (≥99%), and disodium hydrogen phosphate dihydrate (≥99%)—were obtained from Merck (KGaA, Darmstadt, Germany). Acetonitrile and the HPLC-grade methanol solvents were obtained from Merck (KGaA, Darmstadt, Germany); aflatoxin standards B1, B2, G1, and G2 of purity > 98% were obtained from Sigma Aldrich (St. Louis, MO, USA).

### 4.7. Principal Components Analysis (PCA)

The PCA model was generated by applying the algorithm included in the FactoMineR package of the R 4.0.3 Statistical software [33]. For this, the variables were standardized in order to eliminate the influence of scales; later, the PCA algorithm was applied.

## Figures and Tables

**Table 1:** Water activity content, fungal infection, and aflatoxin contamination in maize samples from the Ecuadorian Highlands and Coast.

	Sample	Town or Province	Aflatoxins (µg/kg)					WA *	FI (%) **	Fungal Diversity (% of Ocurrence in the Sample)
			**G2**	**G1**	**B2**	**B1**	**Total**			
Highlands	1	Pichincha	-	-	-	-	-	0.685	8.34	*Cladosporium* sp. (4.17); Dematiaceus (4.17)
	2	Chimborazo	-	-	-	-	-	0.746	100	*A. ruber* (100); *Fusarium* sp. (12.5)
	3	Cotopaxi	-	-	-	-	-	0.689	41.7	Dematiaceus (20.8); *Fusarium* sp. (20.8); *Mucor* sp. (4.17)
	4	Cotopaxi	-	-	-	-	-	0.654	83.4	*A. montevidensis* (4.17); *A. pseudoglaucus* (20.9); *A. restrictus* (12.5); Dematiaceus (4.17); *Fusarium* sp. (37.5);
	5	Carchi	-	-	-	-	-	0.665	nd ***	-
	6	Azuay	-	-	-	-	-	0.647	30.96	*A. pseudoglaucus* (4.17); *A. ruber* (4.17); Dematiaceus (8.37); *Fusarium* sp. (4.17);
	7	Chimborazo	-	-	-	-	-	0.681	54.2	*A. ruber* (37.5); *Fusarium* sp. (8.34); *P. raistricki* (8.34)
	8	Azuay	-	-	-	-	-	0.635	12.5	*Fusarium* sp. (12.5)
	9	Tungurahua	-	-	-	-	-	0.649	54.2	*Fusarium* sp. (54.2)
	10	Azuay	-	-	-	-	-	0.688	100	*A. chevalieri* (4.17); *A. pseudoglaucus* (45.9); *A. ruber* (20.9); *A. wentii* (16.8); Dematiaceus (33.3); *Fusarium* sp. (4.17);
	11	Bolívar	-	-	-	-	-	0.704	100	*A. niger* complex (16.7); *A. ruber* (100); Dematiaceus (12.5); Mucor sp. (12.5)
	12	Chimborazo	-	-	-	-	-	0.720	100	*A. pseudoglaucus* (12.5); *A. ruber* (100); Dematiaceus (16.7); *P. citrinum* (20.8); *Wallemia* sp. (8.30)
Coast	13	Balzar	-	6.34	3.60	97.8	108	0.901	100	*A. Flavi* *** (20.8)*; Fusarium* sp. (79.2); *P. aethiopicum* (41.7)
	14	Ventanas	-	-	-	-	-	0.804	100	***A. Flavi* (8.34)**; *A. wentii* (4.17); *Fusarium* sp. (58.4); *P. citrinum* (95.9)
	15	Portoviejo	-	-	1.81	23.6	25.4	0.839	100	***A. Flavi* (12.5)**; *A. niger* complex (4.17); *Fusarium* sp. (37.5); *P. citrinum* (83.4)
	16	Mocache	-	-	-	-	-	0.821	100	***A. Flavi* (4.17)***; A. chevalieri* (4.17); *Fusarium* sp. (95.9); *P. corylophilum* (4.17)
	17	Empalme	-	-	1.73	25.0	26.8	0.845	95.9	***A. Flavi* (25.0)**; *Fusarium* sp. (54.2); *P. citrinum* (25.0)
	18	Pindal	-	-	-	-	-	0.586	75.1	*A. chevalieri* (12.5); ***A. Flavi* (12.5)**; *A. ruber* (12.5); Dematiaceus (16.6); *Fusarium* sp. (45.9)
	19	Tosagua	-	-	1.73	37.8	39.6	0.861	95.9	*A. Flavi* (12.5)*; Fusarium* sp. (70.9)
	20	Pindal	-	-	-	-	-	0.610	75.06	*A. chevalieri* (4.17); *A. niger* complex (4.17); Dematiaceus (8.34); *Fusarium* sp. (54.2); *T. rugulosum* (4.17)
	21	Balzar	-	-	-	-	-	0.723	75.06	***A. Flavi* (4.17)**; *Fusarium* sp. (70.89);
	22	Tosagua	-	-	-	-	-	0.808	100	***A. Flavi* (16.7)***; Fusarium* sp. (83.4); *P. citrinum* (8.34)
	23	Ventanas	-	-	-	0.42	0.42	0.674	95.9	*A. chevalieri* (33.4); ***A. Flavi* (12.5)**; *A. wentii* (4.17); *Fusarium* sp. (54.2)
	24	Portoviejo	-	-	1.87	29.0	30.8	0.573	100	***A. Flavi* (16.7)**; *A. niger* complex (4.17); Dematiaceus (4.17); *Fusarium* sp. (70.9); *P. citrinum* (25.2)
	25	Mocache	-	-	-	-	-	0.664	100	*A. chevalieri* (8.34); Dematiaceus (8.34); *Fusarium* sp. (62.6)
	26	Empalme	-	-	-	1.50	1.50	0.572	100	***A. Flavi* (25.0)**; *A. niger* complex (4.17); *Fusarium* sp. (70.9)
	27	Pindal	-	-	-	-	-	0.634	75.06	*A. chevalieri* (4.17); *A. niger* complex (4.17); *A. pseudoglaucus* (4.17); *Cladosporium* sp. (8.34); Dematiaceus (8.34); *Fusarium* sp. (41.7); *P. citrinum* (4.17)
	28	Portoviejo	-	-	-	3.43	3.43	0.624	100	*A. chevalieri* (8.34); ***A. Flavi* (20.8)**; *A. wentii* (4.17); Dematiaceus (16.7); *Fusarium* sp. (62.5); *Rhizopus* sp. (4.2)

* WA = Water Activity; ** FI = Fungal Infection; - below the limit of quantification; *** nd = not detected; **in bold** fungi which could produce aflatoxins [6].

## Data Availability

Not applicable.
